# Psychographic Profiling for Effective Health Behavior Change Interventions

**DOI:** 10.3389/fpsyg.2015.01988

**Published:** 2016-01-06

**Authors:** Sarah J. Hardcastle, Martin S. Hagger

**Affiliations:** Health Psychology and Behavioural Medicine Research Group, Faculty of Health Sciences, School of Psychology and Speech Pathology, Curtin UniversityPerth, WA, Australia

**Keywords:** health psychology, behavioral medicine, behavior-change interventions, motivation

Motivating people who are not inclined to engage in health behavior presents a significant challenge to public health practitioners. Although there have been advances in interventions that increase individuals' motivation to engage in health-related behaviors, there is still a relative dearth of evidence as to the processes and mechanisms by which interventions exert effects on behavior. There is also considerable need to identify the moderating factors that enhance or diminish the effects of behavioral interventions. Searching for moderators is important as it will address whether large-scale interventions administered to a population will be effective (a “one size fits all” approach) or whether interventions should be tailored to specific groups with particular demographic, behavioral, and psychological profiles (a “tailored” approach).

Developing effective interventions to facilitate behavior change in health contexts requires a detailed understanding of the demographic, psychological, and ecological factors associated with the behavior. Research has identified multiple correlates of health behavior change, and interventions have been developed to target these factors. Such interventions have shown significant effects in changing behavior, but the size of effects has been modest. A possible reason for the muted effectiveness of interventions is that they do not account for individual differences in salient moderating factors when examining their effects. Target populations in intervention research are rarely grouped according to profiles of these moderators. When segmentation does take place, it is often based on demographic factors such as age, gender, and socio-economic status and not on the psychological and behavioral correlates shown to have pervasive effects on behavior (e.g., Powell et al., 2007; Tapp et al., 2008). As a consequence, individuals recruited for interventions tend to be treated as a homogenous group. However, the assumption of sample homogeneity on variables other than demographics has been recognized as a reason for a lack effectiveness of interventions and the UK Department of Health (2010) has advocated an individualized, tailored approach. In this article, we propose that health behavior change interventions could be more effective if they are tailored to groups of individuals based on key factors likely to moderate the effectiveness of interventions such as motives, preferences, and needs. We argue that improving the effectiveness of interventions for unmotivated individuals should begin with an analysis of the underlying reasons for lacking motivation and support needs when it comes to health behaviors and how these may be specifically targeted in interventions.

The notion of segmentation is effectively used in marketing where it is common practice to distinguish homogenous groups of customers who can be targeted in the same way because they have similar needs and preferences (Wedel and Kamakura, 1998) and is the act of classifying individuals into meaningful sub-groups based on certain characteristics (Wedel and Kamakura, 1998). Social marketing is used to target groups of individuals and to communicate a message tailored to individual needs and preferences (French and Blair-Stevens, 2005). These groupings are based on the premise that people must be treated in different ways because they are motivated by different factors and have different needs. If such segmentation could be applied to health behavior change, intervention developers may be able to target groups with messages tailored to meet the specific characteristics of the segmented groups. There is a need for more psychographic segmentation to reveal patterns or differences between people in a specific cohort. Psychographic segmentation seeks to understand the values, interests and lifestyles of individuals in order to uncover needs and motives (Walker et al., 2014). The identification of distinct sub-groups whose members have unique barriers, motives and needs would assist the development of more tailored and varied interventions. So far, the approach to segmentation has been quantitative a priori or *post hoc* on the basis of multivariate statistical analysis to identify segments. We advocate an alternative approach, and suggest that there is a need to identify groups of individuals with specific profiles of key moderating variables within a sample from the outset and tailor interventions accordingly. The inclusion of psychographic factors adds information about “why” and “what” is needed in addition to the “who” and “where” provided by socio-demographic and geo-demographic information (French et al., 2011).

To date, only a handful of studies have adopted psychographic segmentation applied to health behaviors including physical activity (Boslough et al., 2005; Staten et al., 2006; Sport England, 2009), food choice (Byrd-Bredbenner et al., 2008), and obesity (Wills et al., 2014). However, most approaches to psychographic segmentation have been deductive using surveys to identify clusters (e.g., Boslough et al., 2005; Byrd-Bredbenner et al., 2008; Sport England, 2009). Future research may do well to segment based on inductive, bottom-up methods to ensure that the factors distinguishing clusters of individuals are those voiced by the individuals themselves, rather than factors thought to influence motivation and behavior change, derived from the researcher or interventionist based on theoretical models that assume sample homogeneity. For example, inductively-driven psychographic segmentation by Wills et al. (2014) found that traditional targeting according to the “stages of change” did not fit with people's values and needs. Their more nuanced analysis identified degrees of motivation based on perceived causes and consequences of obesity, barriers and enablers for change that included self-efficacy and social norms. Their segmentation analysis indicated the type of interventions likely to appeal to particular groups who are aware of their obesity but unsure of what to do versus those who were well informed but discouraged by previous unsuccessful weight loss attempts. In light of these findings, and the limitations of top-down approaches to psychographic profiling, future research should, perhaps conduct idiographic psychographic profiling prior to and to inform, the development of more effectively tailored interventions.

In conclusion, we contend that current interventions tend to focus on a generalized, “one size fits all” approach that do not adequately address the different needs of individuals to facilitate change. We propose that researchers and interventionists seek to identify means to segment individuals within their samples based on their motives, preferences, and support needs, and use the evidence to develop more effective, nuanced, and tailored interventions.

## Author contributions

SH conceived the ideas presented in the article and drafted the article. MH helped to draft the article.

### Conflict of interest statement

The authors declare that the research was conducted in the absence of any commercial or financial relationships that could be construed as a potential conflict of interest.

## References

[B1] Boslough S. E. Kreuter M. W. Nicholson R. A. Naleid K. (2005). Comparing demographic, health status and psychosocial strategies of audience segmentation to promote physical activity. Health Educ. Res. 20, 430–438. 10.1093/her/cyg138 15572439

[B2] Byrd-Bredbenner C. Abbot J. Cussler E. (2008). Psychographic segmentation of mothers of young children using food decision influencers. Nutr. Res. 28, 506–516. 10.1016/j.nutres.2008.05.012 19083453

[B3] Department of Health (2010). Maximising the Appeal of Weight Management Services. London: DOH.

[B4] French J. Merritt R. Reynolds L. (2011). Social Marketing Casebook. London: Sage.

[B5] French L. Blair-Stevens C. (2005). Social Marketing Pocket Guide. London: NSMC.

[B6] Powell J. Tapp A. Orme J. Farr M. (2007). Primary care professionals and the social marketing of health in neighbourhoods: a case study approach to identify target and communicate with at risk populations. Prim. Health Care Res. Dev. 8, 22–35. 10.1017/S1463423607000047

[B7] Sport England (2009). Market Segmentation. Available online at: http://segments.sportengland.org/

[B8] Staten L. K. Birnbaum A. S. Jobe J. E. Elder J. P. (2006). A typology of middle school girls: audience segmentation related to physical activity. Health Educ. Behav. 33, 66–80. 10.1177/1090198105282419 16397160 PMC2441540

[B9] Tapp A. Eagle L. Spotswood W. (2008). Social Marketing-Based Strategy for Obesity Interventions. Bristol: University of the West of England (UWE) Available online at: http://eprints.uwe.ac.uk/79

[B10] Walker B. Albertson C. Freeberg R. (2014). Psychographic Segmentation and the Health Care Consumer. Philadelphia: TPG.

[B11] Wedel M. Kamakura W. A. (1998). Market Segmentation: Conceptual and Methodological Foundations. Dordrecht: Kluwer Academic Publishers.

[B12] Wills J. Crichton N. Lorenc A. Kelly M. (2014). Using population segmentation to inform local obesity strategy in England. Health Promot. Int. 30, 658–666. 10.1093/heapro/dau004 24504360

